# Early alterations in hippocampal perisomatic GABAergic synapses and network oscillations in a mouse model of Alzheimer’s disease amyloidosis

**DOI:** 10.1371/journal.pone.0209228

**Published:** 2019-01-15

**Authors:** Jan-Oliver Hollnagel, Shehabeldin Elzoheiry, Karin Gorgas, Stefan Kins, Carlo Antonio Beretta, Joachim Kirsch, Jochen Kuhse, Oliver Kann, Eva Kiss

**Affiliations:** 1 Institute of Physiology and Pathophysiology, University of Heidelberg, Heidelberg, Germany; 2 Institute of Anatomy and Cell Biology, University of Heidelberg, Heidelberg, Germany; 3 Department of Human Biology and Human Genetics, University of Kaiserslautern, Kaiserslautern, Germany; 4 CellNetworks Math-Clinic Core Facility, University of Heidelberg, Heidelberg, Germany; 5 Department of Cellular and Molecular Biology, University of Medicine and Pharmacy of Târgu Mures, Târgu Mures, Romania; Nathan S Kline Institute, UNITED STATES

## Abstract

Several lines of evidence imply changes in inhibitory interneuron connectivity and subsequent alterations in oscillatory network activities in the pathogenesis of Alzheimer’s Disease (AD). Recently, we provided evidence for an increased immunoreactivity of both the postsynaptic scaffold protein gephyrin and the GABA_A_ receptor γ2-subunit in the hippocampus of young (1 and 3 months of age), APPPS1 mice. These mice represent a well-established model of cerebral amyloidosis, which is a hallmark of human AD. In this study, we demonstrate a robust increase of parvalbumin immunoreactivity and accentuated projections of parvalbumin positive (PV+) interneurons, which target perisomatic regions of pyramidal cells within the hippocampal subregions CA1 and CA3 of 3-month-old APPPS1 mice. Colocalisation studies confirmed a significant increase in the density of PV+ projections labeled with antibodies against a presynaptic (vesicular GABA transporter) and a postsynaptic marker (gephyrin) of inhibitory synapses within the pyramidal cell layer of CA1 and CA3. As perisomatic inhibition by PV+-interneurons is crucial for the generation of hippocampal network oscillations involved in spatial processing, learning and memory formation we investigated the impact of the putative enhanced perisomatic inhibition on two types of fast neuronal network oscillations in acute hippocampal slices: 1. spontaneously occurring sharp wave-ripple complexes (SPW-R), and 2. cholinergic γ-oscillations. Interestingly, both network patterns were generally preserved in APPPS1 mice similar to WT mice. However, the comparison of simultaneous CA3 and CA1 recordings revealed that the incidence and amplitude of SPW-Rs were significantly lower in CA1 vs CA3 in APPPS1 slices, whereas the power of γ-oscillations was significantly higher in CA3 vs CA1 in WT-slices indicating an impaired communication between the CA3 and CA1 network activities in APPPS1 mice. Taken together, our data demonstrate an increased GABAergic synaptic output of PV+ interneurons impinging on pyramidal cells of CA1 and CA3, which might limit the coordinated cross-talk between these two hippocampal areas in young APPPS1 mice and mediate long-term changes in synaptic inhibition during progression of amyloidosis.

## Introduction

The pathogenesis of Alzheimer’s disease (AD) is thought to begin much earlier (decades in humans, and months in rodents) than the clinical onset can be diagnosed [1, 2]. Thus, the characterization of specific changes of gene-expression and protein level profiles in presymptomatic stages of mouse models of AD [3, 4] may improve our understanding of the initiation phase of this disease.

A well-established and extensively utilized animal model for AD is the double transgenic APP_swe_/PS1_L166P_ mouse which overexpresses familial AD mutations of human amyloid precursor protein and presenilin-1, resulting in increased Aβ42 levels and thus representing a model of cerebral amyloidosis for AD, with early onset of the amyloid plaque deposition [5]. Recently, we described a biphasic change in the immunoreactivity of several proteins of inhibitory synapses in the hippocampus of APPPS1 mice [4]. Adult transgenic animals (12 months) displayed a remarkable decrease in the level of gephyrin, a postsynaptic organizer of ligand-gated ion channels at inhibitory synapses, in the hippocampal subregions CA1 and dentate gyrus. In contrast, in young, APPPS1 mice (1 and 3 months) we found a robust increase of these proteins as compared to controls. Moreover, the postsynaptic γ2-GABA_A_ receptor subunit and the presynaptic vesicular inhibitory amino acid transporter protein (VIAAT) showed corresponding changes, altogether suggesting a possible increased hippocampal inhibitory drive in the early phase of Aβ—amyloidosis.

Dysfunctions in GABAergic inhibition and the consequent imbalance between excitation and inhibition have been shown to result in hyperexcitability and desynchronisation of neuronal networks [6, 7] leading to impairment of information processing, learning and memory formation [8]. Hence, a better understanding of inhibition, especially in the early pathophysiology of AD is unequivocally pivotal.

The hippocampal neuronal network architecture is ideally suited to provide the framework for generating slow and fast oscillations ranging from very slow to ultra-fast (0.025–600 Hz) [9]. The oscillations in various frequency bands are correlated to different behavioral states. The theta range (4–12 Hz) oscillations are characteristic for explorative behaviour and rapid-eye-movement sleep [10], whereas activity in the γ-range (30–100 Hz) is thought to underlie higher brain functions such as learning, memory and attention [11, 12]. Consummatory behavior, immobility and slow-wave sleep are associated with sharp wave-ripple (SPW-R) complexes (90–200 Hz) [13, 14], which represent brief periods (30–100 ms) of high frequency oscillations of membrane potentials (ripples), co-occuring with large extracellular voltage deflections. SPW-R complexes are thought to be required for memory consolidation [15, 16].

Inhibition of principal cells, either by soma- or dendrite-targeting interneurons, is essential a for sequencing rhythmic activity within the hippocampal network [1]. Interneurons that express the calcium-binding protein parvalbumin (PV) comprise ~26% of the GABAergic neurons in the CA1 region of the hippocampus, and provide the majority of perisomatic inhibitory input onto hippocampal pyramidal cells [18, 19, 20]. These GABAergic synapses play a key role in the generation of both γ-oscillations [17, 21] and SPW-Rs [22] as well as in the control of pyramidal neuron recruitment [23].

Several studies indicate that interneurons and consequently the oscillatory network activities regulated by them are altered in AD [24]. Morphological analysis of human AD post-mortem tissue evidenced a reduction in the immunoreactivity and number of specific subpopulations of Ca^2+^- binding proteins expressing GABAergic interneurons (PV, calretinin, calbindin) in the hippocampus and entorhinal cortex [25, 26, 27, 28] though less alterations or even maintenance of these cells were detected in some cortical regions [29, 30, 31]. Assessments of brain sections of symptomatic AD-model-mice provided similar findings [32, 33, 34]. Reduced γ-band power and synchronization has been recorded in symptomatic AD-patients [35, 36]. Similarly, adult hAPP-expressing AD-mice had reduced γ-activity at baseline and diminished inductions during exploratory activity [37, 38]. Dysfunction of PV+ interneurons by depletion of the Nav1.1 channel resulted in impaired oscillatory rhythms and desynchronisation in hippocampal circuits of adult AD-mice [38]. In addition, the frequency and temporal structure of SPW-Rs in CA1 were disrupted in a transgenic mouse model of dementia (rTg4510) at an age of established neurodegeneration and cognitive impairment [39]. However, the cellular mechanisms that cause changes of oscillatory network activities, in particular during the early stages of AD, are poorly understood.

Our previous observations of an increased level and preferential somatic localization of gephyrin in the CA1 pyramidal cell layer of the hippocampus of 1- and 3-month-old APPPS1 mice raised the question whether these early changes coincide with alterations in the connectivity of PV+ interneurons. Thus, the aim of the present study was to gain insight in further potential changes of inhibitory synapses at an early stage of amyloidosis, prior to the formation of senile plaques and significant cognitive loss in APPPS1 mice. Considering our former findings of significant and consistent changes primarily at the age of 3 months, here we investigated 3-month-old APPPS1 mice and corresponding WT littermates (I) to monitor PV-immunoreactivity and density in comparison to key pre- and postsynaptic proteins of inhibitory synapses in hippocampus by fluorescence microscopy and immunoblotting and (II) to characterize properties of SPW-Rs and γ-oscillations monitoring indirectly the effects of inhibitory perisomatic synaptic transmission to pyramidal cells in areas CA3 and CA1 of acute hippocampal slices. Our results provide evidence for an increased GABAergic synaptic output of PV+ interneurons onto the pyramidal cell layer in CA1 and CA3 of the hippocampus of 3-month-old APPPS1 mice and suggest a disturbed synchrony between these two hippocampal areas during network activities already at this early stage of amyloid pathology.

## Material and methods

### Animals

In this study 3-month-old male APPPS1 mice and age-matched nontransgenic littermates (WT) were used. All animal procedures were performed in accordance with the European Communities Council Directive (86/609/EEC) and were approved by the responsible regional state authorities of Baden-Württemberg (T-65/15 and G-72/17).

APPPS1 mice were obtained from Prof. Dr. M. Jucker (University of Tübingen, Germany) and bred in the animal unit of the University of Kaiserslautern. Offspring was genotyped as described elsewhere [5, 40]. The double transgenic APPPS1 mice coexpress the KM670/671NL „Swedish”mutated amyloid precursor protein (APP) and the L166P-mutated human presenilin 1 (PS1) under the control of a neuron-specific Thy1 promoter element on a C57BL/6 background. This model shows initial amyloid plaque deposition at 2 months of age in the neocortex and at 4–5 months in the hippocampus. The ratio of β-amyloid42 to β-amyloid40 is 1.5 in young (1-month-old) and ~5 in amyloid-depositing older mice, respectively [5]. Adult mice exhibit abnormal tau phosphorylation but lack intraneuronal tau deposition (neurofibrillary tangles) and show local but not global neuronal loss [41]. Cognitive impairment, including deficits in the Morris Water maze test was observed at seven months of age [42]. Impairment of LTP in the hippocampal area CA1 has also been reported to start around this age [40].

### Tissue preparation

Mice (4–6 animals/group) were deeply anesthetized with isoflurane and perfused transcardially with phosphate buffered saline (PBS) (pH 7.4) (Sigma Aldrich, Steinheim, Germany) (~10 ml) followed by 4% paraformaldehyde (PFA, ~50 ml). The animals were decapitated and the brains were prepared. Brain hemispheres were separated, transferred to 4% (w/v) PFA for 1h (at 4°C) rinsed in PBS and cryoprotected by immersion in 10% (w/v) (30 min), 20% (w/v) (1h), and 30% (w/v) sucrose (overnight). The samples were mounted in OCT embedding compound (VWR Chemicals, Leuven, Belgium) and snap-frozen on an absolute ethanol-dry ice mixture. For biochemical analysis fresh hippocampus was dissected from unfixed brain hemispheres and immediately frozen in liquid nitrogen. Tissue samples were stored at −80 °C until use.

### Immunolabeling

Coronal cryostat sections (8 μm) cut from fresh-frozen brains were mounted on SuperFrost Plus slides (Menzel GmbH, Braunschweig, Germany) and stored at -20°C. They were then thawed, fixed with 4% (w/v) PFA for 8 min and preincubated for 20 min at 95 °C in sodium citrate buffer (10 mM, 0,05% Tween-20, pH 6.0) for antigen retrieval. After three rinses in PBS (pH 7.4) and preincubation in blocking solution (5% normal horse serum, 5% bovine serum albumin, 0,2% TritonX-100 (Roth, Karlsruhe, Germany) for 60 min, sections were incubated overnight with primary antibodies diluted in blocking solution without TritonX-100. Individual sections were incubated with up to three primary antibodies (for colocalisation of antigens), detected with secondary antibodies conjugated to fluorophores (Vector Laboratories, Invitrogen, Jackson Immunoresearch Laboratories) and mounted with mounting medium Mowiol/Dabco (Roth, Karlsruhe, Germany). The following primary antibodies were used: monoclonal rabbit or mouse anti-parvalbumin (1:500, Swant PV235 or 1:200, Merck Millipore, Darmstadt, Germany), polyclonal guinea pig or polyclonal goat anti-vesicular GABA transporter (VGAT) (1:1000, Synaptic Systems, Göttingen, Germany, or D-18 1:50, Santa Cruz Biotechnology), polyclonal chicken anti-glutamate decarboxylase (GAD) 67 (1:1000, Abcam) and monoclonal mouse anti-gephyrin, clone mAb7, phosphor-specific at AA S270 (1:500, Synaptic Systems, Göttingen, Germany and see [43]. DAPI (4′,6-diamidino-2-phenylindole, 1:200) was used to visualize nuclear DNA. To reduce autofluorescence tissue sections were treated with „Autofluorescence Eliminator Reagent”(Merck Millipore, Darmstadt, Germany) according to the manufacturer`s recommendations. Controls omitting the primary antibodies were included. Serial sections from APPPS1 and WT mice were labelled simultaneously using the same batches of solutions to avoid differences in immunolabeling conditions.

### Confocal laser scanning microscopy and quantitative immunofluorescence analysis

Confocal microscopy was performed with a Leica TCS SP8 microscope (Leica Microsystems CMS GmbH, Mannheim, Germany) using a HC PL APO CS2 63.0 × 1.40 oil objective as described in detail previously [4]. Briefly, the emission filter settings were 490–540 nm for PMT2 (green), 555–620 nm for PMT3 (red) and 630–665 nm for PMT4 (cyan). The images were acquired in sequential mode with a frame average of 4. Stacks of 8 optical sections (1024x1024 pixels) spaced by 500 nm were recorded. 4 randomly chosen fields within CA1 and 3 fields within CA3 of each hippocampus (n = 4–6 brains/group) were recorded for quantitative analysis. In each field, rectangular areas of 600x150 μm within the pyramidal cell layer (PCL) were randomly selected and measured. Laser power and settings were identical for all samples in an experiment. The recorded images were quantified for mean fluorescence intensities and number of colocalized immunofluorescent puncta (density) using NIH’s Fiji (pacific.mpi-cbg.de/wiki/index.php/fiji). Mean fluorescence intensity of a region of interest was calculated from maximal intensity projection of 8 optical sections. To analyze the colocalization of inhibitory synaptic components (gephyrin, VGAT) with PV+ buttons in the PCL of CA1 and CA3 areas an ImageJ/Fiji macro was developed (CellNetworks Math-Clinic Core Facility, University of Heidelberg, Germany) which first semi-automatically segmented the immunofluorescent puncta using the threshold method and then automatically processed the generated binary masks to find overlapping signals between the three different confocal channels. The number of puncta were then counted by the ImageJ/Fiji macro and a customized summary table was created in the output directory for each processed image for further validation and statistical analysis. Mean values calculated for each animal were used for final statistics. WTs were set to 1, and data are given as mean ± SEM [4].

The total number of interneurons stained positively for PV (the PV label filling entire cells) was counted in 3–5 fields/section in 3–5 sections/brain, 4–5 brains/group for each region of interest, and a mean value/ field was calculated.

### Protein extracts and immunoblot analysis

The protocols were described in detail previously [4]. Briefly, frozen hippocampus was homogenized in 350 μl ice-cold lysis buffer: 50 mM Tris-HCl, pH 7.5, 150 mM NaCl, 1% (v/v) nonyl phenoxylpoly-ethoxylethanol (NP40), 0.25% (w/v) sodium dodecyl sulfate (SDS), 2 mM Na-orthovanadate and protease inhibitor cocktail (cOmplete, Roche) using PrecellysR homogenization tubes (91-PCS-CK14S) with two intervals of maximal shaking intensities (60s), each in a tissue mixer mill (MM301, Retsch). Extracts were passed through a cannula (0.4 x 19 mm) (10 x fold) and centrifuged (30 min, 15000xg; 4°C) before total lysates were used for protein determination using a BCA-assay kit (Thermo Scientific). 30 μg of total protein extracts were adjusted to 1 x Laemmli-loading buffer conditions (2% SDS, 10% glycerol, 5% 2-mercaptoethanol, 0.002% bromphenol blue and 0.06M Tris HCl, pH 6.8.), heated to 95°C for 5 min, loaded onto 12.5% polyacrylamid-SDS gels, and separated with constant voltage. Proteins were blotted on polyvinylidene difluoride membranes (Millipore) according to the manufacturer’s instructions. Membranes were probed with mouse anti-parvalbumin antibody (1:500, BD Transduction Laboratories, 610495) and mouse anti-β-actin antibody (1:20000, Abcam, AC-15, ab6276). The corresponding horseradish peroxidase-conjugated secondary antibodies (Bio-Rad) were detected using ECL Prime detection kit (Amersham Biosciences). After exposure to hyperfilms (Amersham Bioscience) pixel intensities of the bands of interest were analyzed using ImageJ. Immunoreactive band intensities were normalized to the corresponding signals revealed by anti-β actin antibodies.

### Acute slice preparation

3-month-old APPPS1 and WT littermates were used (n = 5 animals/group). Mice were decapitated during isoflurane anesthesia. To prevent temporary blood-brain barrier opening [44], the amount of and exposure to isoflurane was reduced by dissolving 1.5 vol% of the narcotic in a gas mixture comprising of 70% N_2_O and 30% O_2_. Following decapitation, brains were immediately transferred into artificial cerebrospinal fluid (aCSF, ~ 4°C; see below) saturated with 95% O_2_ and 5% CO_2_. Horizontal hippocampal slices (400 μm) were prepared using a Leica VT1000S Vibratome (Wetzlar, Germany). Slices were immediately transferred in an interface type recording chamber, perfused with aCSF (saturated with 95% O_2_ and 5% CO_2_) at a flow rate of 1.8 ml/min and maintained at 36°C. Slices prepared from each animal (WT and APPPS1) were distributed within two recording chambers.

For each recording session, one animal of each group was sacrificed and up to 3 slices per animal were used. Experimenters were blinded to the type of mice.

### Recording solution and drugs

Acute slices were constantly supplied with pre-warmed (36°C) aCSF that contained (in mM): 129 NaCl, 21 NaHCO_3_, 1.25 NaH_2_PO_4_, 1.8 MgSO_4_, 1.6 CaCl_2_, 3 KCl, 10 glucose (Sigma-Aldrich). The osmolarity was 300 ± 5 mOsmol/l and pH was 7.4 when saturated with the ambient gas mixture (95% O_2_ and 5% CO_2_). Recordings of acute slice preparations were started around 2h after preparation.

Following a stable baseline recording of at least 10 minutes during SPW-Rs, persistent γ-oscillations were induced by bath application of the cholinergic agonist carbachol (CCh, 10 μM; Sigma-Aldrich).

### Electrophysiology

Extracellular local field potentials (LFP) from CA3 and CA1 of WT and APPPS1 animals were recorded in parallel in AC mode under interface conditions. We used carbon fiber electrodes (Kation Scientific, Minneapolis, MN, USA; 0.4–1.2 MΩ) and glass electrodes (filled with aCSF, 5–10 MΩ) pulled from GB150F-8P borosilicate capillaries (Science Products GmbH, Hofheim, Germany) using a vertical micropipette puller (DMZ Zeitz-Puller, Zeitz-Instruments Vertriebs GmbH, Martinsried, Germany). LFPs were amplified using an EXT 10-2F amplifier in an EPMS-07 housing (npi Electronic GmbH, Tamm, Germany), filtered at 3 KHz, digitized on-line at 10 kHz (CED-1401, Cambridge Electronic Design, Cambridge, United Kingdom) and stored on a computer disk with Spike2 (Cambridge Electronic Design) for offline analysis.

### Data analysis

Offline analysis was performed in MatLab (MathWorks, Natick, MA, USA) using custom written routines. To analyze SPW-Rs, signals were first separated into their slow (wave) and fast components (ripples). The slow component is obtained by low-pass filtering (FFT filter, cut frequency: 80 Hz) and used for event detection and calculation of amplitude and duration. The ripple component was isolated by a band-pass filter (FFT filter, pass-band frequency: 120–400 Hz). Ripples were counted only when subsequent ripples crossed a threshold of 3 times the standard deviation (SD) of the band-pass filtered signal. For analysis of γ-oscillations data segments of 5 min were subdivided into pieces of 30 sec length, band-pass filtered (FFT filter, pass-band frequency: 5–200 Hz) and processed with Welch’s algorithm and a fast Fourier transformation (FFT size: 8192). The resulting power spectral density (PSD) plots had a resolution of 1.2207 Hz. γ-oscillations were analyzed for various parameters, i.e., peak power spectral density (pPSD), peak frequency (f), area under the curve (AUC), full width at half maximum (FWHM) and inner coherence (TAU) as described in detail earlier [45]. Medians of subdivisions were calculated and used for further statistical analysis. Data are summarized by their median ± the interquartile range (IQR = 75% percentile—25% percentile), if not stated otherwise. Number of animals (n) and preparations (N) are provided in the Figs that summarize the experiments. We accepted only slices displaying SPW-Rs on average higher than 0.04 mV and occurring at least with 1 Hz. Recordings in the presence of carbachol were accepted when the peak frequency was higher than 25 Hz.

### Statistical analysis

Statistical evaluation was done in Prism (GraphPad Software Inc., La Jolla, CA, USA). Statistical significance of immunofluorescent and immunoblot data were determined using unpaired Student’s t test or Mann Whitney test as indicated in the Fig legends: * = p<0.05, ** = p<0.01 and *** = p<0.001. Numeric values are given as mean ± standard error of the mean (SEM) or standard deviation (SD) as indicated in the figure legends.

For electrophysiology, if data were normally distributed (Shapiro-Wilk), statistical evaluation was performed by a one-way analysis of variance (ANOVA) with Holm-Šídák’s correction for multiple comparisons to identify significant differences between more than two conditions. Non-parametric tests (Kruskal–Wallis) were used and followed by Dunn’s multiple comparisons, when data were not normally distributed. p-values less than 0.05 were considered to indicate a significant difference between groups (indicated by asterisks).

## Results

In a previous study, we observed increased immunoreactivity of the GABAergic postsynaptic marker gephyrin in perisomatic regions of the pyramidal cell layer (PCL) of the CA1 region of young (1- and 3-month-old) APPPS1 mice, possibly corresponding to early stages of cerebral amyloidosis in AD [4]. As most inhibitory projections onto the soma and proximal dendrites of excitatory pyramidal neurons come from PV+ interneurons [40], we analyzed here the extent of PV+-projections onto the PCL in both the CA1 and CA3 region of the hippocampus in 3-month-old APPPS1 mice by immunohistochemistry and by immunoblotting and compared to age-matched WT littermates. Furthermore, we evaluated parvalbumin-related fast network oscillations in acute hippocampal slices of these animals.

### Increased PV+ neuronal projections in hippocampal subregions CA1 and CA3 of young APPPS1 mice

**Fig 1:**
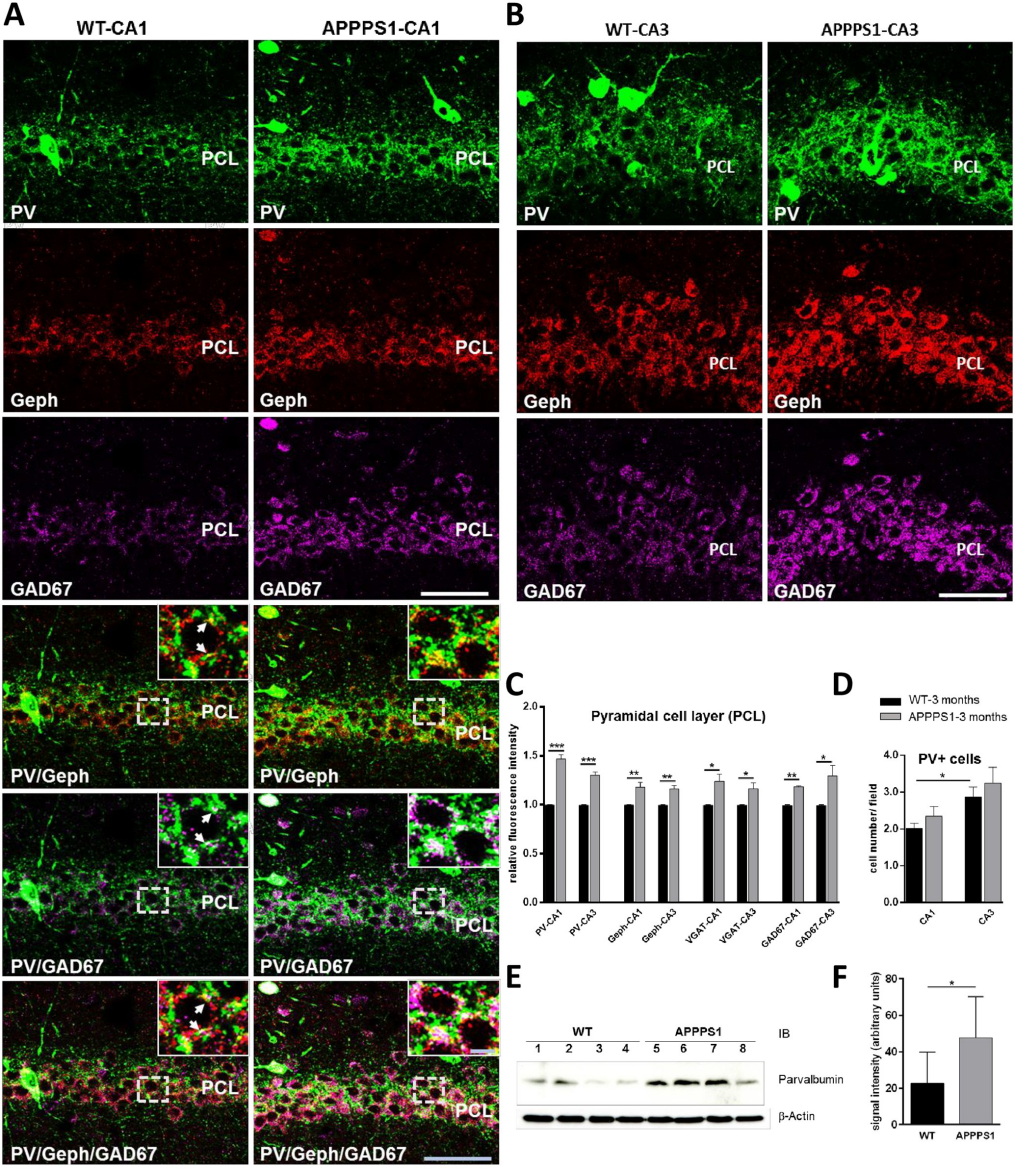
Increased PV immunoreactivity (IR) in CA1 and CA3 hippocampal subregions of 3-month-old APPPS1 mice. (A) Triple-immunolabeling of perfusion-fixed cryosections for parvalbumin (green), gephyrin (red), and GAD67 (magenta). Single channel acquisitions show the increased density of PV+ projections, gephyrin-, and GAD67-IR in the pyramidal cell layer (PCL) of CA1 (A, upper panel) and CA3 (B). Merged images demonstrate higher numbers of overlapping gephyrin or/and GAD67 immunoreactivities on PV+ projections on unstained somata of cells in CA1 PCL in APPPS1 hippocampus compared to WT (A, lower panel). Arrows in insets indicate the PV/gephyrin, PV/GAD67 double-, respectively triple-positive structures. (A, B): Confocal maximum intensity projections (8 optical sections, 3,5-μm thick z-stack); Scale bars: 50 μm; insets: 5 μm. (C): Quantification of mean immunofluorescence intensities (see Methods) for PV and marker proteins of inhibitory synapses (gephyrin, VGAT, GAD67) in the PCL of CA1 and CA3 areas of hippocampus of WT and APPPS1 mice (measurements were done in PV+ cell free regions of PCL; n = 4–6 animals/group; WTs were set to 1 and data are given as means +/- SEM; *p < 0.05, **p < 0.01, ***p < 0.001; Student t-test. (D). Bar graphs indicate the number of PV+ somata quantified in the PCL and stratum oriens (SO) of CA1 and CA3 subregions of the hippocampus (quantified in 3–5 fields/section in 3–5 sections/brain, 4–5 brains/group for each region) in WT and APPPS1 mice. Means +/- SEM; *p < 0.05; Student t test (E): Representative immunoblots of hippocampal lysates obtained from 3-month-old WT and APPPS1 mice and probed with anti-PV antibody and a mouse anti-β-actin antibody. (F) Quantification of protein band intensities shown in E. Note the significant increase of PV protein levels in the APPPS1 mice. Quantification of band intensities was done for four mice for each genotype (n = 4). Mean ± SD. Student t-test, *p<0.05.

**Fig 2:**
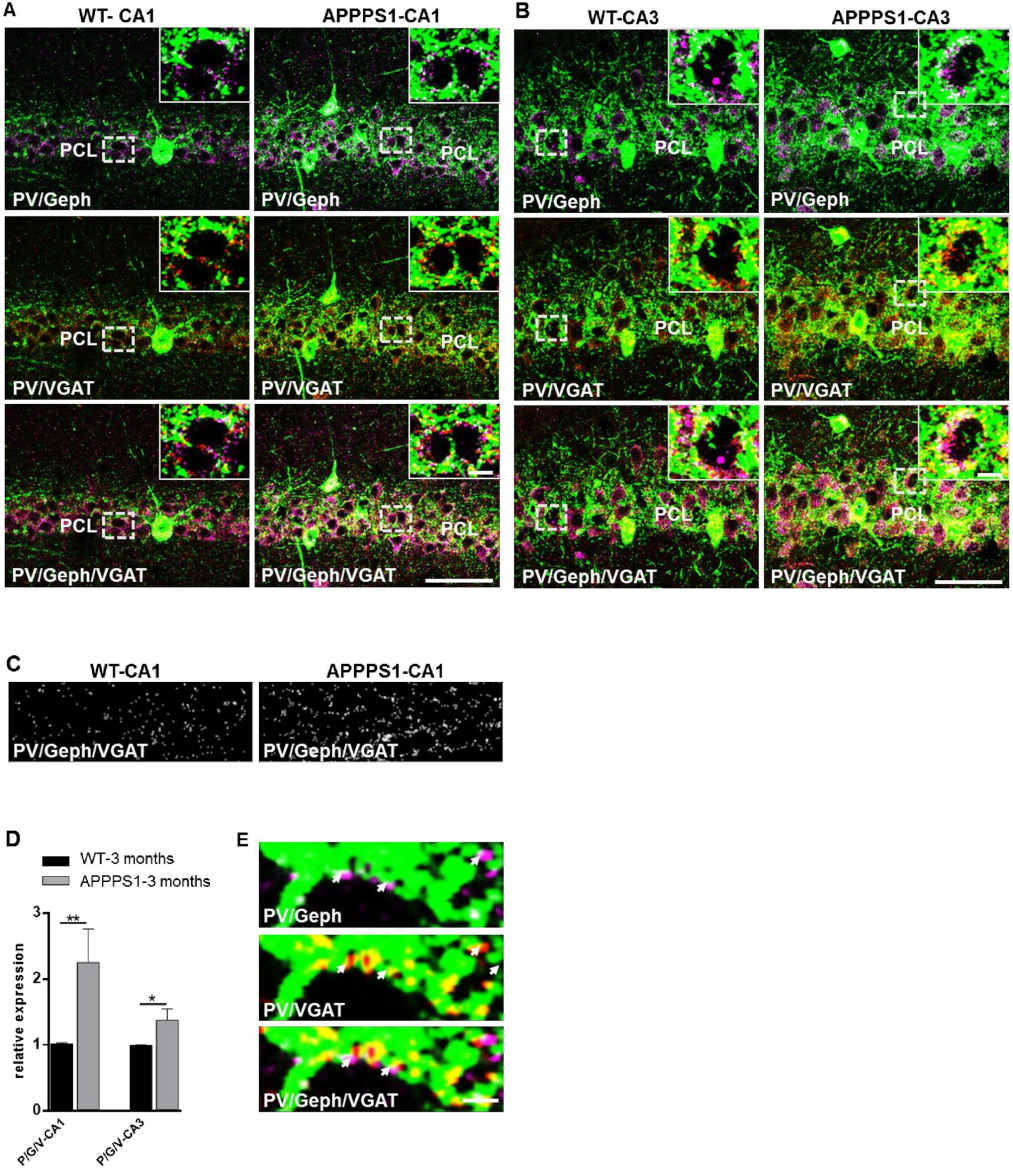
Increased number of triple (VGAT-/gepyhrin-/ PV) immunopositive inhibitory synaptic boutons in PCL of hippocampal CA1 and CA3 subfields of young APPPS1. Images from perfusion-fixed cryosections triple-immunolabeled for parvalbumin (PV) (green), gephyrin (Geph) (magenta), and VGAT (red). (A). Merged images illustrate the increased numbers of gephyrin or/and VGAT clusters along PV+ projections in the PCL of CA1 (A, upper panel) and CA3 (B) areas of hippocampus in 3-month-old APPPS1 mice in comparison to WT. (A, B): Confocal maximum intensity projection images (8 optical sections, 3,5-μm thick z-stack); Scale bars: 50 μm and 5 μm (insets). (C). Images show triple-(VGAT/Geph/ PV) positive puncta as detected by the analysis program (see Methods) in randomly selected areas within the PCL of WT and APPPS1 hippocampus. (D). Graph bars show the increase in the number of triple (VGAT/Geph/PV) labelled clusters in the PCL of CA1 and CA3 hippocampal regions in APPPS1 in comparison to WT. (n = 3 animal/group; 5 fields/brain for CA1 and 3 fields/brain for CA3); WTs were set to 1, and data are given means +/- SEM; *p < 0.05; **p < 0.01, Mann-Whitney test. (E). High magnification images show perisomatic PV+ projections (green) with Geph (magenta) and VGAT (red) immunoreactivities largely overlapping (arrows) as well as puncta positive for a single marker only. Confocal images of 1 optical section (0,5 μm thick); Scale bar: 2 μm.

In 3-month-old APPPS1 mice the overall pattern of PV immunoreactivity was comparable to that seen in brains of age-matched WT animals. The majority of PV+ interneurons was located within the stratum pyramidale (PCL) and stratum oriens (SO). Additionally, PV+ fibers generated a densely clustered, granular plexus around the somata and proximal dendrites of pyramidal cells within the PCL (Fig 1A), representing a major fraction of the somato-dendritic inhibitory input onto these cells. However, in comparison to WT, PV immunoreactivity was increased in the hippocampus of APPPS1 mice as shown for CA1 in Figs 1A and 2A and for CA3 in Figs 1B and 2B. Quantification of fluorescence intensities using confocal images (4 and 3 fields/section for CA1 and CA3, respectively; n = 4–6 brains/group) revealed that in the hippocampus of APPPS1 mice the overall intensity of PV immunoreactivity in the PCL was significantly higher (~45% increase in CA1; ~30% increase in CA3) compared to WT brains (Fig 1C). The number of PV+ somata (PV+ interneurons) in the PCL and SO was higher in CA3 compared to CA1, both in wildtype and APPS1 mice, although, a trend to a higher number was seen in the sections of the APPPS1 hippocampus (Fig 1D).

To verify the GABAergic nature of the PV+ terminals in the PCL of CA1 and CA3 we used triple immunolabeling with antibodies directed against gephyrin, the postsynaptic scaffold protein of GABA_A_ receptors and to GAD67, the key enzyme for the synthesis of the inhibitory neurotransmitter GABA. The analysis revealed that the increase in PV immunoreactivity paralleled with an increase in GAD67 and gephyrin immunoreactivities in the PCL of both CA1 (Fig 1A) and CA3 (Fig 1B) of APPPS1 mice. Postsynaptic gepyhrin clusters often colocalized with GAD67+ immunoreactivities, which probably represented presynaptic boutons [46] on PV+ perisomatic projections (Fig 1A). These findings were confirmed by measurements of fluorescence intensities in confocal sections (in PV+ somata—free regions) (Fig 1C) as well as by semiquantitative analysis of immunoblots of protein extracts from hippocampus of APPPS1 and WT mice (Fig 1F and 1G). Our results suggest a functionally important sprouting of PV+ terminals in the hippocampal subregions of 3-month-old APPPS1 mice.

### Increased number of inhibitory synaptic clusters on PV+ projections in the PCL of CA1 and CA3 subregions of young APPPS1 hippocampus

To enable reliable identification of presynaptic release sites of PV+ processes we used a triple immunostaining for parvalbumin, gephyrin and the presynaptic marker VGAT. Our results confirmed increased relative fluorescence intensity of gephyrin clusters and a moderate increase in the relative fluorescence intensity of VGAT terminals in the PCL (Fig 1C). As shown in Fig 2A and 2B most PV+ perisomatic projections exhibited VGAT immunoreactivities, indicating presynaptic terminals of the GABAergic interneurons, which were closely opposed to gephyrin-positive puncta, representing the corresponding inhibitory postsynaptic sites. In addition to VGAT and gephyrin clusters largely overlapping (not only on PV+ projections), puncta positive for only one of the markers were also seen (Fig 2A, 2B and 2E), reflecting the particular heterogeneity within the population of inhibitory synapses of the hippocampal subfields. To determine the numerical density of synapses established by PV+ interneurons the colocalisation of gephyrin and VGAT immunoreactivities on PV+ projections in randomly selected areas of the PCL was quantified (see Methods). The density of PV+/VGAT+/gephyrin+ clusters was significantly higher in CA1 (~70%) as well as in CA3 (~35%) of the APPPS1 hippocampus in comparison to WT (Fig 2C and 2D), indicating an increased PV-specific GABAergic somatodendritic input on pyramidal cells in the hippocampus of 3-month-old APPPS1 mice.

### Subregion-dependent increase in the immunoreactivity of PV and inhibitory synaptic proteins

**Fig 3:**
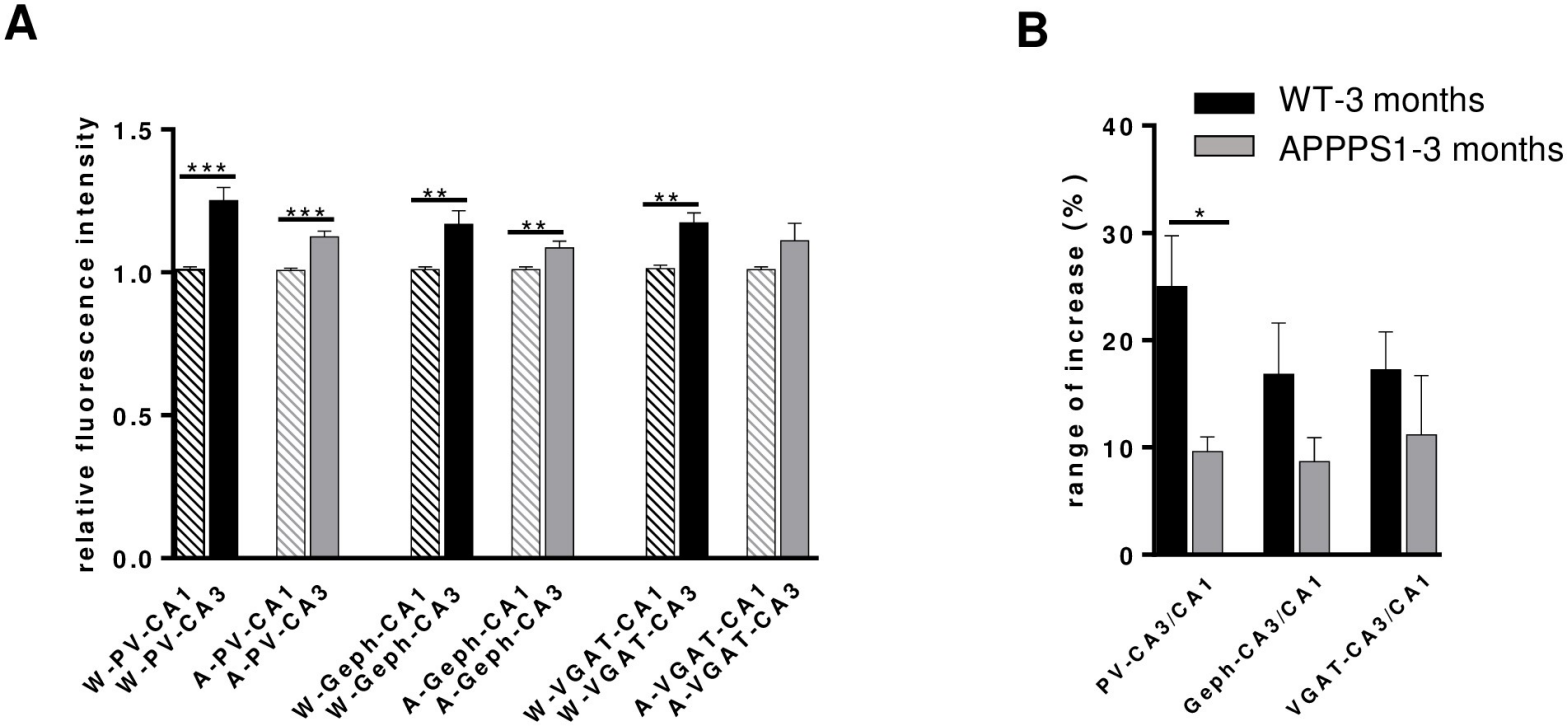
Subregional differences in the immunoreactivity of PV and inhibitory synaptic proteins in the hippocampus of 3-month-old WT and APPPS1 mice. (A): Graphs show in comparison the immunofluorescence intensities measured for PV, Geph and VGAT in CA1 and CA3 subfields of WT and APPPS1 hippocampus, respectively. The immunoreactivity of all three antigens is higher in CA3 than in CA1 both in WT and APPPS1 mice. However, the rate of increase in CA3 vs CA1 is significantly higher in APPPS1 hippocampus (B). The interregional difference CA3 vs CA1 in PV-, Geph- and VGAT-immunoreactivity in WT and APPPS1 hippocampus is given as percent (%) when CA1 was set as 100% (n = 4–5 animals/group; means +/- SEM; *p < 0.05).

Comparison of the CA1 and CA3 subregions revealed that the immunoreactivity of the PV+ meshwork in PCL was significantly denser in the CA3 than in the CA1 area in both WT and APPPS1 mice, as was the density of PV+ somata occurring in the PCL and SO of CA3 and CA1 (Fig 1D). However, the significant difference between CA3 and CA1 of ~ 25% in WT mice was, in spite of the overall increase in PV immunoreactivity, diminished in APPPS1 mice to a level of about ~ 12% (Fig 3A and 3B). These results not only confirm a regional difference in the distribution pattern of PV in the mouse hippocampus, observed also by other groups [33, 34], but point to an altered balance between CA3-CA1 hippocampal subregions in the 3-month-old APPPS1 mice. This observation was less prominent for other markers of inhibitory synapses (VGAT, gephyrin) (Fig 3A and 3B), which might partially rely on the notorious aldehyde sensitivity of the relevant epitopes of these proteins [47, 48]. However, perfusion-fixation of the brains was necessary to optimize the detection of parvalbumin.

### Hippocampal network oscillations indicate an altered balance between CA3 and CA1 in young APPPS1 mice

Individual PV+ interneurons in CA1 and CA3 project to a large number of neighboring pyramidal neurons thereby coordinating hippocampal oscillations and patterning neuronal ensembles [49]. To elucidate whether the putative increased inhibitory input from PV-interneurons in the hippocampus of 3-month-old APPPS1 mice might change network oscillations, we recorded local field potentials simultaneously in hippocampal areas CA3 and CA1 of acute slices prepared from APPPS1 and WT animals. In mouse hippocampal slices, SPW-Rs emerge spontaneously in CA3 and propagate to CA1 [50]. They represent the default network state in the present study. The spontaneously occurring SPW-Rs were recorded for at least 10 min. Subsequently, we recorded persistent γ-oscillations in the presence of carbachol (10 μM).

### Sharp wave-ripple complexes

**Fig 4:**
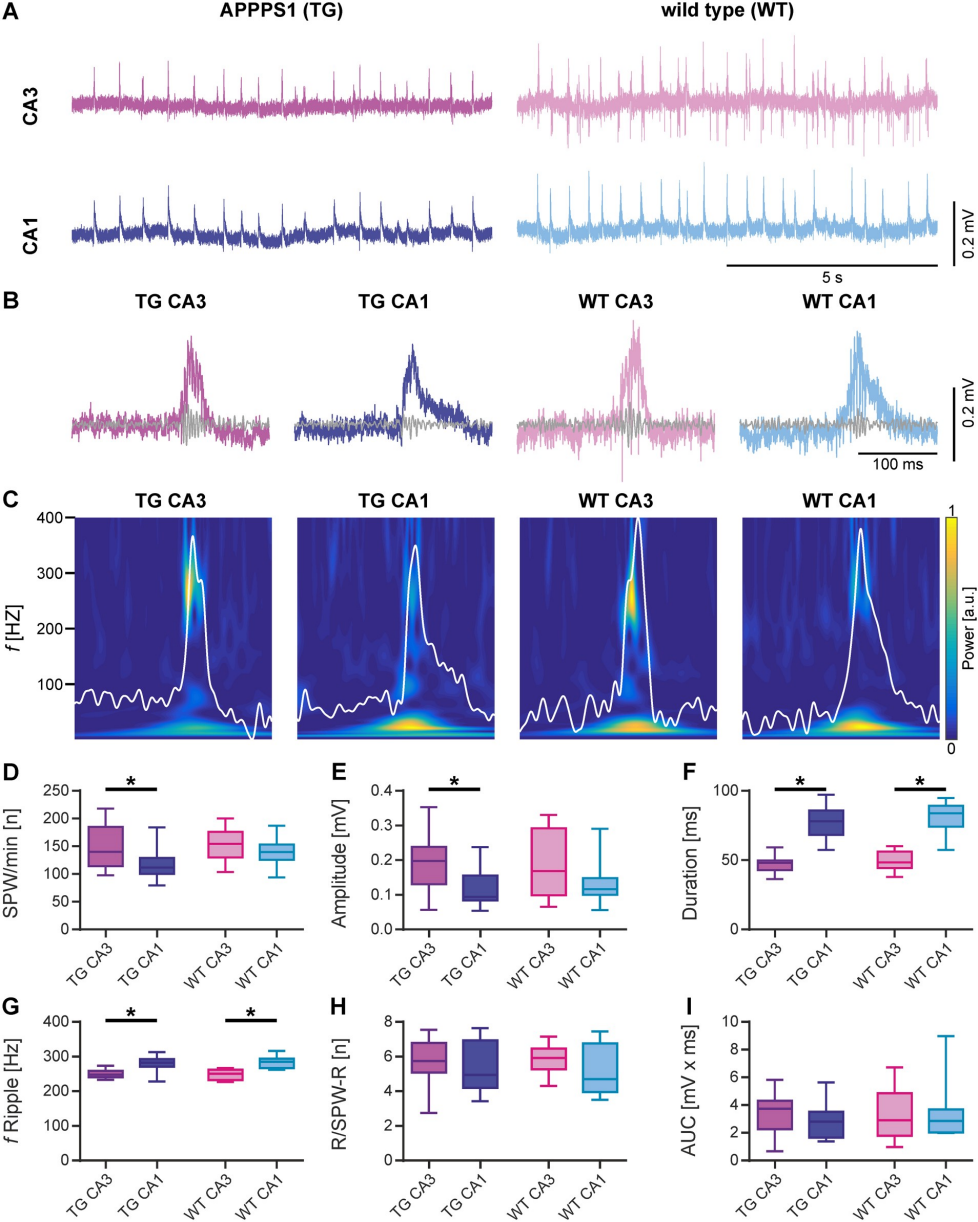
Comparison of spontaneous sharp wave-ripple activity in acute hippocampal slices from transgenic APPPS1 (TG) and wild type (WT) mice. (A) Sample LFP traces for paired recording of hippocampal areas CA3 and CA1 from APPPS1 (left) and WT (right) animals. (B) Representative examples of single SPW-R complexes. (C) Wavelet transformation of the same SPW-Rs shown in B representing the power of frequency domains over time. Color represents power from low (blue) to high (yellow). The slow component of each SPW-R (low-pass: 80 Hz) is plotted in white. (D-I) SPW-Rs were analyzed for different parameters. (D) Number of SPW-Rs per minute (SPW/min). The number of SPW-Rs was significantly lower in CA1 compared to CA3 in APPPS1 animals. (E) Amplitude of SPW-Rs. The amplitude was significantly lower in CA1 compared to CA3 in transgenic animals. (F) Duration of SPW-Rs. SPW-Rs were significantly longer in CA1 compared to CA3 in both WT and transgenic animals. (G) Ripple frequency of SPW-Rs (f Ripple). Ripple frequencies were significantly faster in CA1 compared to CA3 in both WT and transgenic animals. (H) Number of ripples per SPW-R complex (R/SPW-R). (I) Area under the curve (AUC) of SPW-Rs. Data are represented by median (horizontal line within box) and interquartile range (IQR; with the box ranging from first to third quartile). The whiskers indicate minimum and maximum of data (APPPS1 CA3 & APPPS1 CA1, N = 5, n = 15; WT CA3 & WT CA1, N = 5, n = 10). Asterisks denote significance (p < 0.05) against CA3 for APPPS1 and WT animals, respectively.

To examine the impact of a putatively increased inhibitory drive in APPPS1 mice, we first compared key features of SPW-Rs in CA3 and CA1 areas of hippocampus. As indicated by representative examples in Fig 4A–4C, SPW-Rs were reliably detected within each group (APPPS1 and WT) and in both hippocampal areas. In WT mice, the incidence (Fig 4D) and amplitude of SPW-Rs (Fig 4E) was quite similar among areas CA3 and CA1. The duration of SPW-Rs (Fig 4F) and the frequency of ripples (Fig 4G), however, was significantly higher in CA1 than in CA3 (WT CA3 vs. WT CA1; duration: p<0.0001, frequency of ripples: p<0.0001). Interestingly, the number of ripples per sharp wave was similar in both areas (Fig 4H) as was also the area under the curve of the slow component (Fig 4I).

As opposed to WT mice, the incidence of SPW-Rs (Fig 4D) was significantly lower in CA1 of APPPS1 mice compared to CA3 (APPPS1 CA3 vs. APPPS1 CA1; p = 0.0183) and the amplitude (Fig 4E) was significantly lower in CA1 compared to CA3 (APPPS1 CA3 vs. APPPS1 CA1; p = 0.0201). The duration of SPW-Rs (Fig 4F) as well as the frequency of ripples (Fig 4G), similarly to the WT, was significantly higher in CA1 than in CA3 (APPPS1 CA3 vs. APPPS1 CA1; duration: p<0.0001, frequency of ripples: p<0.0001). The number of ripples per sharp wave (Fig 4H) and the area under the curve of the slow component (Fig 4I) were similar in CA3 and CA1.

While most features of SPW-Rs were not affected in APPPS1, we found significantly less numerous and powerful SPW-Rs in CA1 as compared to CA3, suggesting that an increase in inhibition confines the intrahippocampal communication between CA3 and CA1.

### γ-oscillations

**Fig 5:**
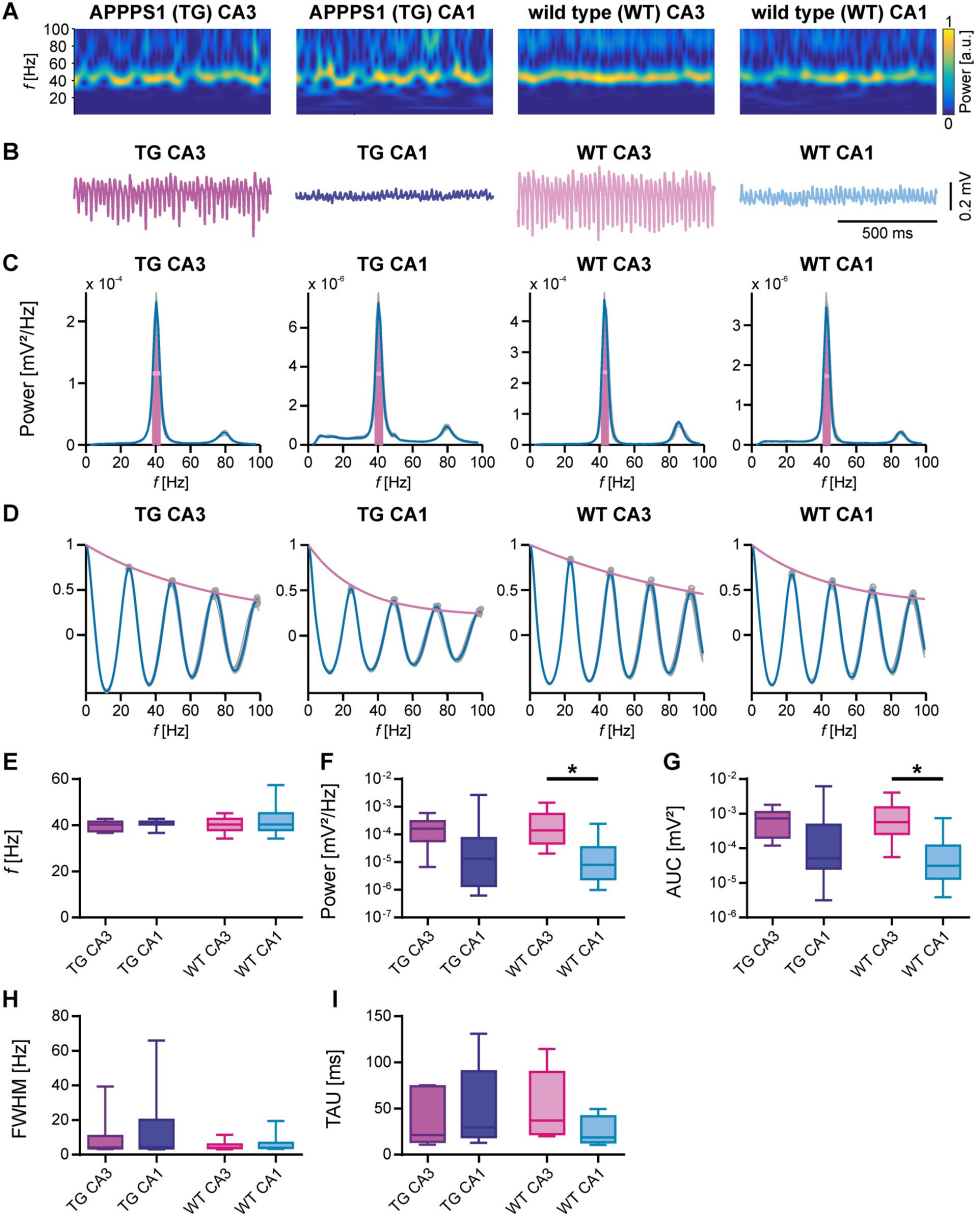
Comparison of carbachol-induced γ-oscillations in acute hippocampal slices from transgenic APPPS1 (TG) and WT mice. (A) Wavelet transformation of sample LFP traces shown in B representing the power of frequency domains over time. Colour represents power from low (blue) to high (yellow). (B) Sample LFP traces (5–200 Hz filtered) of γ-oscillations induced by 10 μM carbachol recorded simultaneously in areas CA3 and CA1 of APPPS1and WT animals. (C) Power spectra derived from the last 5 min of each recording shown in B (median of 10 windows, 30 sec duration). The horizontal line (pink) represents the full width at half maximum (FWHM). The area under the curve (AUC) is shown in magenta. (D) Autocorrelations derived from the last 5 min of each recording shown in B (median of 10 windows, 30 sec duration). The time constant (TAU, see I) was derived from the exponential fit (magenta) to the peaks of the autocorrelation. (E-I) γ-oscillations were analysed for different parameters. (E) Peak frequency (*f*). γ-oscillations were maximal at around 40 Hz in CA3 and CA1 of APPPS1 and WT animals. (F) Peak of power spectral density (pPSD). Power of γ-oscillations was generally lower in CA1 than in CA3. The difference was significant in WT animals. (G) Area under the curve (AUC). AUC of power spectra was generally lower in CA1. The difference was significant in WT animals. (H) Full width at half maximum (FWHW) showed higher variability in APPPS1 mice as in WT mice. (I) Time constant (TAU). TAU derived from autocorrelations was not significantly different between subregions and/or mice strains. Data are represented by median (horizontal line within box) and interquartile range (IQR; with the box ranging from first to third quartile). The whiskers indicate minimum and maximum of data (APPPS1 CA3 & APPPS1 CA1, N = 5, n = 8; WT CA3 & WT CA1, N = 5, n = 11). Asterisks denote significance (p < 0.05) against CA3 for APPPS1 and WT animals, respectively.

Following the period of SPW-R activity, we induced γ-oscillations in the very same slices by bath application of carbachol (10 μM) and further quantified amplitude, power, distinctness and coupling strength between CA3 and CA1. As indicated by the corresponding spectrograms (Fig 5A) of the representative LFP traces (Fig 5B), γ-oscillations could be reliably induced in transgenic as well as in WT animals. Corresponding power spectra of γ-oscillations (Fig 5C) were used for calculation of frequency, power, area under the curve and full width at half maximum, while autocorrelations (Fig 5D) were used to calculate the time constant TAU.

The frequency of γ-oscillations (Fig 5E) was not significantly different among areas CA3 and CA1 in WT mice. However, the corresponding power (Fig 5F) was significantly lower in CA1 compared to CA3 (WT CA3 vs WT CA1; p = 0.0086). Accordingly, the area under the curve (Fig 5G) of the power spectra was also significantly lower in CA1 than in CA3 (WT CA3 vs WT CA1; p = 0.0027). The other parameters analysed to describe γ-oscillations (full width at half max and the time constant; Fig 5H and 5I) were similar within hippocampal areas CA3 and CA1 of WT mice.

In contrast, in APPPS1 mice no substantial difference in the power of γ-oscillations between CA1 and CA3 could be detected. As shown in Fig 5E–5I in APPPS1 hippocampal slices, all parameters of γ-oscillations (Fig 5E–5I) were not significantly different between CA3 and CA1.

This finding suggests that the increased inhibition present in the 3-month-old APPPS1 mice decreases the distinctness of γ-oscillations between CA3 and CA1. γ-oscillations in area CA1 appear to be better tuned by the increased inhibitory drive found in the 3-month-old APPPS1 mice.

## Discussion

Our study demonstrates a striking increase in synaptic projections of GABAergic PV+ interneurons in CA1 and CA3 regions of 3-month-old APPPS1 mice. This finding supports the hypothesis of GABAergic dysregulation in the pathogenesis AD [51]. Moreover, our results depict PV+ interneurons as effectors of early changes during the amyloidogenic process of AD, prior to formation of amyloid plaques.

Recently, we observed a robust increase in the expression of both postsynaptic γ2-GABA_A_ receptor subunits and gephyrin, the key scaffold protein of inhibitory synapses, in the CA1 and DG subregions of the hippocampus of 3-month-old APPPS1 mice compared to age matched WT mice. Moreover, we demonstrated an increase in expression and synaptic patterning of phosphorylated gephyrin [4], which regulates GABA_A_R clustering [52, 53], Here, we confirmed the increase in gephyrin immunoreactivity and provide evidence for a corresponding increased number and fluorescence intensity of PV+-interneuron projections in hippocampal subregions CA1 and CA3 of the of 3-month-old APPPS1 mice. Although the density of PV+ processes was boosted in APPPS1 hippocampus, the number of PV+ interneurons was not appreciably increased compared to WT. This is in concordance with recent studies showing enhanced immunoreactivity but no or little change in the number of PV+ cells in the hippocampus and entorhinal cortex of relative young (3–6 months) APPPS1 mice [38, 54, 55]. Quantification of overlapping PV/VGAT/gephyrin immunoreactivities in triple immunolabeled brain sections disclosed a statistically significant increase in the density of perisomatic GABAergic synapses originating from PV+ interneurons and terminating around pyramidal cells in both CA1 and CA3 subregions of the hippocampus of APPPS1 mice as compared to WT. Further, the higher number of PV+ inhibitory synaptic immunoreactivities was paralleled by an increased expression of GAD67, the main GABA synthesizing enzyme. These data are in line with the findings of Bell and coworkers who observed an increased density of GAD65-positive presynaptic boutons in the cortex of a 4-month-old TgCRND8 AD-mouse-model [56] and support an enhancement of GABAergic neuronal function. Thus, our morphological data point to an increased somato-dendritic inhibitory input of PV+ interneurons in CA1 and CA3 subregions of the hippocampus of young APPPS1 mice.

PV+ interneurons are known to highly interconnect with one another and to create a vast synchronously active cell network, which control the firing properties of pyramidal cells as detailed in previous works [47, 20]. They are considered to play a key role in triggering and maintaining memory-related network activity patterns such as γ- oscillations and SPW-Rs in the hippocampus and other brain regions [49]. Several studies provide evidence for an impairment of γ-oscillations in advanced stages of AD with deficient frequency, power and amplitude, which might be attributed to reduced inhibitory drive, due to age related loss of PV-interneurons [32] or reduced activity of these cells [58]. However, little is known about the functional changes upon an increased inhibitory drive. Optogenetic stimulation studies in wild type mice showed generation of γ-oscillatory activity upon specific activation of PV+ cells [21, 59]. Mice with neuron-targeted expression of the presynaptic gain-of-function glycine receptor RNAvariant GlyR α3L185L to genetically enhance the network activity of PV+ interneurons displayed increased SPW-R activity in hippocampal areas CA3 and CA1 and facilitated propagation of this particular network activity pattern as determined in hippocampal slice preparations and described in detail previously [60]. In addition, in Goto-Kakizaki (GK) rats with type 2 diabetes,—a metabolic disorder with elevated risk of AD—and severe memory problems, a significant increase in PV+ interneuron density in hippocampal CA3/CA4 areas and enlargement of the somatic volume of PV+ interneurons in CA1/CA2 areas was coupled with a shift from slow to fast γ-range oscillations, while the frequency variance of the oscillations decreased, probably caused by a strengthened synchronization of phasic inhibition [61].

In this context, we analyzed spontaneous SPW-Rs and carbachol-induced γ-oscillations, and compared measurements of CA3-CA1 network activities using slices from 3-month-old transgene- and WT-mice. Interestingly, general properties as incidence, frequencies, amplitude and duration of SPW-Rs as well as amplitude and power of γ-oscillation were very similar in APPPS1 compared to wild type mice. However, when comparing CA1 vs CA3 of the same strain, we found that the intrahippocampal differences in the incidence and amplitude of SPW-Rs and the power of γ-oscillations are altered in the APPPS1 mice in comparison to WT.

SPW-Rs in the hippocampus are self-organized activity patterns that emerge from the extensive recurrent excitatory collaterals within area CA3 [13] under the control of inhibitory interneurons [62]. In the CA3 generated SPW-Rs yield spatiotemporally synchronized spiking in CA1 with the potential goal to amplify the output messages of the hippocampus [57]. Thus, the precise interplay between the excitatory and inhibitory processes during ripples may aim to rapidly select the dominant and suppress the competing cell ensembles and thereby drive forward temporally organized and strongly synchronous messages to downstream cortical and subcortical structures as described in detail previously by Stark et al [57]. Based on these data one might speculate that in our study an increase in inhibition might limit the communication between CA3 and CA1 areas of the hippocampus as suggested by the increased difference in the incidence of SPW-Rs in transgenic but not in WT-animals. In this context, it has to be noticed that the enhanced inhibitory synaptic density in the APPPS1 mice as assessed by both fluorescence intensities for PV and number of triple labeled inhibitory synapses was unequal for the CA1 and CA3 subregions, with a much more robust increase in CA1. Thus, the morphological data correspond with the results of the electrophysiological analysis showing an increased difference in the incidence and amplitude of SWR-Rs between CA1 and CA3 areas in APPPS1 mice in comparison to WT and probably represent one morphological correlate to the electrophysiological outputs. The findings might fit to the hypothesis, that amyloid β affects distinct types of synapses, neurons and brain regions and even subregions to varying extents. These differential changes could further contribute to imbalances and instabilities [33, 63]. Trinchese et al reported that associative long-term synaptic potentiation (LTP), a form of synaptic plasticity is attenuated in the CA1 subregion of an APPPS1 (APP_Swe_ PS1_M146L_) mice at 3–4 months of age which correlated with elevated levels of Aβ and abnormal short memory [64]. In the same mouse model by whole-cell patch clamp recordings Viana de Silva et al detected abolished LTP in CA3 pyramidal cells at 6 months of age [65] Additionally, Klein and coworkers using an *in vitro* model of kainate induced γ-oscillations detected reduced γ-frequency and power in the lateral entorhinal cortex (LEC) of 4–5-month-old APPPS1 (APP_Swe_/PS1_L166P_) mice but not in the medial entorhinal cortex (MEC) [55], altogether underscoring the spatio-temporal differences in vulnerability of the different brain regions along AD-pathogenesis.

Thus, despite a substantial elevation in the number of inhibitory synaptic terminals originating from PV+ interneurons in the PCL of the hippocampus of 3- month-old APPPS1 mice we did not see major qualitative or quantitative alterations in SPW-Rs and γ- oscillations in the hippocampal slices of APPPS1 mice in comparison to WT. These findings denote that upregulation of PV+GABAergic terminals detected in our study marking the early-stage of β-amyloidosis in the APPPS1 mice cannot be directly compared to data recorded in the presence of an increased firing rate of PV+ cells in WT mice or via genetically enhanced activity of PV+ interneurons [21, 59, 60].

Given the absence of major changes in the two PV-dependent hippocampal network oscillations the adjustment of inhibitory innervation in the hippocampus of 3-month-old APPPS1 mice is most probably a homeostatic mechanism, which compensates for the activity changes among hippocampal cell populations and aims to balance activity to an appropriate level that permits functions in a physiological range. Especially soluble Aβ species (i.e., monomeric, oligomeric, and protofibrillary) are thought to initiate the disease process in AD by impairing structural and functional plasticity of excitatory synapses and leading to a compensatory upregulation of glutamatergic and cholinergic presynaptic boutons during early stages of the amyloid pathology, as observed in transgenic mouse models of AD and in patients with mild cognitive impairment [56, 66, 67]. Moreover, soluble toxic species of Aβ can induce reactive astrocytosis,—a frequent finding in both human and mouse model AD-brain-, and cause, as recently shown for reactive astrocytes in the DG of APPPS1 mice, GABA release from these cells which could contribute to the excitatory-inhibitory imbalance in AD [68, 69]. The GABAergic sprouting in the APPPS1 mice should enhance synaptic inhibition in order to compensate aberrant increases in network excitability as described for the dentate gyrus in hAPP mice [70]. In fact, structural synaptic plasticity is considered a core mechanism of homeostatic plasticity and is associated with structural remodeling of excitatory and inhibitory synapses [71, 72]. Earlier immunohistochemical studies on human post-mortem tissue demonstrated increased axonal and dendritic sprouting and synaptogenesis in the neocortex and hippocampus of AD-patients and also in normal aging [73, 74], with regional and temporal differences [75] indicating that this form of synaptic plasticity might be an early feature of AD, preceding detectable neurofibrillary tangle formation and extensive neuronal loss [76, 77]. Recent in vivo experiments revealed that inhibitory synapses are also dynamic structures and can undergo continuous rearrangements [72, 78]. Further, homeostatic synaptic plasticity can adjust neuronal firing rates [79]. Specifically, the work of Flores et al. has revealed that synaptic and neuronal activity can directly regulate the number and function of perisomatic inhibitory synapses through a mechanism that involves the phosphorylation of gephyrin [52]. These findings, as previously described in detail [43], hint to a mechanism, which may be crucially important to individually optimize the level of inhibition on pyramidal neurons and, thus, set the proper balance required for the synchronization of oscillations mediated by parvalbumin interneurons during learning and memory [17, 21, 80]. Furthermore, the data point to our recent findings (as mentioned above) upon the significant increase of phosphorylated gephyrin in CA1 and DG in the APPPS1 mice [4] as a compensatory mechanism and underscore the critical role of PV+ interneurons in the early phase of AD amyloidosis.

Although most appropriate, the increase in density of inhibitory synapses might not be occurring simply as a compensatory mechanism. Some alterations might be generated by the direct effect of Aβ on GABAergic synapses [66, 81]. Most recently, Chen et al have shown, that APP can physically interact with KCC2, a neuron-specific K^+^-Cl^-^ cotransporter that is essential for Cl^-^ homeostasis and fast GABAergic inhibition in hippocampus [82]. Although, in a model of AD of intracerebroventricular administration of soluble Aβ_1–42_ which leads to memory impairment and loss of nerve terminals whithin 2 weeks the density of GABAergic terminals (VGAT positive) was not affected in the hippocampus of C57BL6 mice [83]. In addition to APP, presenilin 1 might also be involved in the alterations of inhibitory synapses observed in our study on APPPS1 mice. Besides contributing to Aβ generation as catalytic subunits of γ-secretase, there is growing evidence that presenilins play an essential role in the formation and maintenance of synapses. PS1 single transgenic animals present enhanced dendritic spine density of cortical and hippocampal CA1 neurons and at an age of 3–5 months evidence increased GABA_A_ transmission relative to excitatory transmission [84]. This effect seems to be γ-secretase-independent, but rather related to altered Ca^2+^ -homeostasis. Dysregulated Ca^2+^ -homeostasis has been linked to Alzheimer’s pathology at various levels [85]. The increased immunoreactivity of the calcium buffer protein parvalbumin in the hippocampus of 3-month-old APPPS1 mice is in agreement with an earlier report of Verdaguer and coworkers [54] that points to the activation of signaling cascades towards hippocampal calcium homeostasis maintenance at early stages of AD. Calcium, in return orchestrates homeostatic synaptic adaptations [86]. Interestingly, the mode of synaptic adaptation in the hippocampus seems to be rather heterogeneous under different etiological conditions leading to memory impairment. In a model of chronic stress associated with decreased performance in memory tests immunocytochemistry of purified individual nerve terminals revealed a selective increase in the number of glutamatergic rather than of GABAergic (VGAT)-positive) terminals in the hippocampus of mice exposed to 3 weeks of unpredictable stress [87]. The same group using a bred-based model of depression, associated with memory dysfunction detected a reduction in the density of VGAT positive presynaptic but not of gephyrin positive postsynaptic terminals in hippocampal synapses of 11-12-week-old „helpless”mice [88]. Though, none of these studies detailed the subregional distribution or particular changes in PV+ synaptic projections which would offer more insight in representative correlations concerning hippocampal inhibitory synaptic plasticity in memory-related alterations. Finally, it should be noted that our data refer to the APPPS1 mice which co-express mutations of both APP and PS1 genes associated with the familial forms of AD and which promote overproduction of Aβ accelerating cerebral amyloidosis in these mice, but do not model the tau pathology and robust neurodegeneration observed in AD [5, 41]. Some of the phenotypes observed in our study might be the result of APP- or APP/PS1-overexpression and might not reflect the slowly progredient course of sporadic AD pathology. However, this mouse model is unequivocally useful for studying effects of soluble Aβ species on the various early stage aspects associated with AD.

In summary, our findings support a compensatory upregulation of hippocampal inhibitory GABAergic terminals and underscore the crucial role of plastic changes of PV+ interneurons [89] during the early state of Aβ overproduction in AD maintaining initially subtle the alterations at the network level.
